# Risk of longer-term endocrine and metabolic conditions in the Deepwater Horizon Oil Spill Coast Guard cohort study – five years of follow-up

**DOI:** 10.1186/s12940-025-01164-9

**Published:** 2025-03-22

**Authors:** Hristina Denic-Roberts, Lawrence S. Engel, Jeanine M. Buchanich, Rachel G. Miller, Evelyn O. Talbott, Dana L. Thomas, Jordan McAdam, Jill E. Emerick, Tina Costacou, Jennifer A. Rusiecki

**Affiliations:** 1 Department of Epidemiology, School of Public Health, University of Pittsburgh, Pittsburgh, PA USA; 2 Oak Ridge Institute for Science and Education, Bethesda, MD USA; 3 Department of Epidemiology, Gillings School of Public Health, University of North Carolina, Chapel Hill, NC USA; 4 Department of Biostatistics, School of Public Health, University of Pittsburgh, Pittsburgh, PA USA; 5 United States Coast Guard Headquarters, Directorate of Health, Safety, and Work Life, Washington, D.C USA; 6 The Henry M. Jackson Foundation for the Advancement of Military Medicine, Inc, Bethesda, MD USA; 7 Department of Preventive Medicine and Biostatistics, Uniformed Services University of the Health Sciences, Bethesda, MD USA; 8 Department of Pediatrics, Uniformed Services University of the Health Sciences, Bethesda, MD USA

**Keywords:** Deepwater Horizon Oil Spill, Crude oil, Dispersants, U.S. Coast Guard spill responders, Endocrine conditions, Metabolic conditions

## Abstract

**Introduction:**

Long-term endocrine and metabolic health risks associated with oil spill cleanup exposures are largely unknown, despite the endocrine-disrupting potential of crude oil and oil dispersant constituents. We aimed to investigate risks of longer-term endocrine and metabolic conditions among U.S. Coast Guard (USCG) responders to the *Deepwater Horizon* (DWH) oil spill.

**Methods:**

Our study population included all active duty DWH Oil Spill Coast Guard Cohort members (*N* = 45,224). Self-reported spill exposures were ascertained from post-deployment surveys. Incident endocrine and metabolic outcomes were defined using *International Classification of Diseases* (9th Revision) diagnostic codes from military health encounter records up to 5.5 years post-DWH. Using Cox proportional hazards regression, we estimated adjusted hazard ratios (aHR) and 95% confidence intervals (CIs) for various incident endocrine and metabolic diagnoses (2010–2015, and separately during 2010–2012 and 2013–2015).

**Results:**

The mean baseline age was 30 years (~ 77% white, ~ 86% male). Compared to non-responders (*n* = 39,260), spill responders (*n* = 5,964) had elevated risks for *simple and unspecified goiter* (aHR = 2.09, 95% CI: 1.29–3.38) and *disorders of lipid metabolism* (aHR = 1.09, 95% CI: 1.00–1.18), including its subcategory *other and unspecified hyperlipidemia* (aHR = 1.10, 95% CI: 1.01–1.21). The *dysmetabolic syndrome X* risk was elevated only during 2010–2012 (aHR = 2.07, 95% CI: 1.22–3.51). Responders reporting ever (*n* = 1,068) vs. never (*n* = 2,424) crude oil inhalation exposure had elevated risks for *disorders of lipid metabolism* (aHR = 1.24, 95% CI: 1.00–1.53), including its subcategory *pure hypercholesterolemia* (aHR = 1.71, 95% CI: 1.08–2.72), the *overweight, obesity and other hyperalimentation* subcategory of *unspecified obesity* (aHR = 1.52, 95% CI: 1.09–2.13), and *abnormal weight gain* (aHR = 2.60, 95% CI: 1.04–6.55). Risk estimates for endocrine/metabolic conditions were generally stronger among responders reporting exposure to both crude oil and dispersants (vs. neither) than among responders reporting only oil exposure (vs. neither).

**Conclusion:**

In this large cohort of active duty USCG responders to the DWH disaster, oil spill cleanup exposures were associated with elevated risks for longer-term endocrine and metabolic conditions.

**Supplementary Information:**

The online version contains supplementary material available at 10.1186/s12940-025-01164-9.

## Introduction

The 2010 explosion of the offshore drilling rig *Deepwater Horizon* (DWH) caused the largest marine oil spill in U.S. history [1, 2]. Fresh crude oil flowed into the Gulf of Mexico for 87 days, from April 20th until July 15th, 2010, when the head of the well was successfully capped [1]. According to various estimates, 185 to 210 million gallons of crude oil were spilled into the Gulf waters during the DWH spill [1–5]. Two oil dispersants approved by the U.S. Environmental Protection Agency (EPA), COREXIT 9527A and 9500A, were applied on and below the water’s surface to rapidly disperse the spilled oil [1]. It has been estimated that approximately 1.8 million gallons of dispersants were applied during the spill response [1]. While many federal agencies participated in the DWH spill cleanup efforts, the U.S. Coast Guard (USCG) led the national interagency response and deployed approximately 8,700 service members to assist in the cleanup [1]. The main operational cleanup phase lasted through December 17th, 2010. Thousands of workers and volunteers who participated in the cleanup efforts, as well as people residing along the Gulf coast, were potentially exposed to numerous toxic chemicals, including those in crude oil and oil dispersants, which may have adversely impacted their health.

Crude oil contains thousands of chemicals including volatile organic compounds (VOCs), polycyclic aromatic hydrocarbons (PAHs), hydrogen sulfide, benzene, and heavy metals [6]. COREXIT dispersants also contain several different chemical compounds including 2-butoxyethanol, propylene glycol, light weight petroleum distillates, dioctyl sodium sulfosuccinate (DOSS), as well as proprietary substances [7–9]. Some of the constituents of crude oil and oil dispersants may have endocrine disrupting properties. In fact, in vivo and in vitro exposures to PAHs have been associated with disruption of endocrine function through mechanisms of estrogen receptor activation, steroidogenesis, lipogenesis, thyroid signaling disruption, anti-estrogenic activity, and anti-androgenic activity [10–12]. Additionally, it has been demonstrated that DOSS has obesogenic properties, [13, 14] which could also adversely impact the endocrine system. Despite the biological plausibility of endocrine disruption, studies examining endocrine disrupting health effects associated with exposures following oil spill cleanup activities are sparse [15–18]. A handful of investigations, all following the 2002 *Prestige* oil spill off the coast of Northwestern Spain, have studied sub-clinical endocrine outcomes and observed acute [15–17] and longer-term [18] alterations in endocrine blood biomarkers among exposed oil spill cleanup workers and volunteers. In all four studies, plasma prolactin and cortisol levels were measured as potential biomarkers of endocrine toxicity [15–18]. Compared to a non-exposed group, mean plasma prolactin levels were slightly higher among the exposed spill cleanup participants in both cross-sectional [16] and prospective [18] investigations. However, in contrast to the initial cross-sectional findings, mean cortisol levels were significantly higher among the exposed group than the non-exposed group seven years post-spill [18]. As the study investigators noted, one of the main limitations of studying measurements of plasma cortisol and prolactin at a single time point is that these potential endocrine toxicity markers may also be affected by other exposures, such as urban pollution and stress [18]. The Gulf Long-term Follow-up Study (GuLF Study) is the only study to date to have investigated a relationship between an oil spill cleanup exposure and risk of a clinical endocrine disease up to six years post-spill [19]. In this prospective investigation of DWH spill cleanup workers/volunteers, there was a significant association between increasing levels of an estimated total hydrocarbon exposure levels and elevated type 2 diabetes risk among overweight individuals (p-trend = 0.03), but not in the overall study population (p-trend = 0.22) [19].

There have been no other published studies investigating associations between oil spill exposures and other clinical outcomes that could be related to the endocrine system disruption, such as overweight and obesity, metabolic syndrome, lipid abnormalities, and thyroid disorders. Because of the aforementioned potential endocrine-disrupting properties of crude oil and oil dispersant constituents, understanding the relationship between oil spill exposures and endocrine health is of critical importance for preventing endocrine and metabolic damage among people who will be involved in cleanup of future oil spills and those residing in proximity of future oil spill disasters. The aim of our study was to investigate the risk of longer-term endocrine and metabolic conditions among USCG responders to the DWH oil spill in a well-established cohort study, the Deepwater Horizon Coast Guard (DWH-CG) Cohort [20].

## Materials and Methods

### Study Population and Study Design

Our study population originated from the DWH-CG Cohort, which has been described previously [20]. For the present prospective cohort study, we included only the 5,964 responders (i.e., were ordered to respond to the DWH oil spill for at least one day) (68.6%) and 39,260 non-responders (i.e., did not respond to the oil spill) (87.6%) who were on active duty, because only active duty military service members, and not Selected Reservists, have comprehensive medical coverage through the Military Health System (MHS). Inclusion and exclusion criteria for the current study are summarized in Supplemental Fig. 1. More detailed information about the MHS, a healthcare system designed for equal access, was described previously [21]. We obtained ongoing health encounter data for querying endocrine and metabolic diagnoses for all active duty cohort members.

This study was approved by the Institutional Review Boards (IRB) of the Uniformed Services University (USU), the USCG, and the University of North Carolina, Chapel Hill. A waiver for informed consent was approved by the USU IRB.

### Exposure Ascertainment

To examine the exposure of spill response work overall, we compared all active duty spill responders (*n* = 5,964) to non-responders (*n* = 39,260). Responder status was ascertained from USCG administrative databases. The remaining exposure metrics were based on the oil spill response and were, thus, applicable to responders only (i.e., within-responder comparisons). Self-reported exposures to crude oil/oily water (hereafter referred to as “crude oil”) and oil dispersants (hereafter referred to as “dispersants”) were ascertained from two post-deployment exit surveys, described previously [20]. The median length of time between end of deployment and survey completion was one day for Survey 1 and 185 days for Survey 2. The two surveys evaluated similar exposures; however, Survey 1 was more concise. Both surveys included questions about crude oil exposure via inhalation, skin contact, ingestion, and submersion, but Survey 1 evaluated these exposures on a binary scale (i.e., “never/ever”) while Survey 2 evaluated these exposures on a 5-point Likert scale (never, rarely, sometimes, most of the time, and all of the time). Additionally, only in Survey 2 was self-reported exposure related to coming into contact with dispersants ascertained, also on a 5-point Likert scale. In total, 3,492 active duty responders completed at least one of the two surveys, though 390 completed only Survey 1 and hence had no information on dispersants.

For the within-responder comparisons, we evaluated several survey-based exposure metrics: crude oil exposure via 1) inhalation, 2) direct skin contact, and 3) a combined crude oil/dispersants exposure metric. For the crude oil exposure metrics, we combined responses from the two post-deployment surveys and compared DWH responders (*n* = 3,492) who reported “ever” exposure to crude oil to those reporting “never” exposure. For the crude oil exposures via inhalation and via direct skin contact, we combined “ever” responses from Survey 1 and "sometimes," "most of the time,” and "all of the time" responses from Survey 2 into an exposed category “ever” and compared it to a non-exposed category “never” combining "never" responses from Survey 1 with "never" and "rarely" responses from Survey 2. For the exposure metric including a combination of crude oil and dispersants exposures (*n* = 3,102 responders), we created the following exposure groups: “neither” (i.e., reported “never” exposure to crude oil via any route *and* “never” exposure to dispersants); “oil only” (i.e., reported “ever” exposure to crude oil via any route *and* “never” exposure to dispersants); and “both” (i.e., reported “ever” exposure to crude oil via any route *and* exposure to dispersants of “rarely”, “sometimes,” “most of the time,” or “all of the time”). We used the “neither” group as the reference category for the comparisons with “oil only” and “both”. Because there were few responders who reported any exposure to dispersants but no exposure to crude oil (i.e., < 10 responders reported exposure to dispersants only), we did not create a “dispersants only” exposure category.

### Outcome Ascertainment

Health encounters that included endocrine and metabolic conditions were queried from the MHS Data Repository (MDR), a medical health encounter data repository maintained by the U.S. Department of Defense, described previously [20–22]. Briefly, the MDR contains information from inpatient and outpatient health encounters occurring in both military treatment facilities and clinics (“direct care”) and civilian treatment facilities for which care is billed to the military (“purchased care”). We combined MDR data from inpatient and outpatient direct and purchased care sources between October 1, 2007 (~ 2.5 years pre-DWH oil spill) and September 30, 2015 (~ 5.5 years post-DWH oil spill).

During our study period all of the health encounter MDR diagnoses were coded using the Ninth Revision of the *International Classification of Diseases* (ICD-9) codes. We evaluated diagnoses of chronic endocrine and metabolic diseases and symptoms classified by three-, four-, or five-digit ICD-9 codes. We considered individual and grouped ICD-9 codes for various endocrine and metabolic conditions including thyroid disorders, disorders of parathyroid, pituitary, and adrenal glands, diabetes mellitus, disorders of lipid metabolism, gout, metabolic syndrome, overweight and obesity, abnormal weight gain, and impaired glucose tolerance. A full list of individual and grouped conditions we evaluated, along with the corresponding ICD-9 codes, is provided in Supplemental Table 1. Our incident case definition for classifying endocrine and metabolic outcomes required having at least one inpatient or two outpatient encounters/visits with a specific individual endocrine/metabolic condition or a group of endocrine/metabolic conditions in any diagnostic position. Prevalent cases were defined as those who had a pre-existing endocrine/metabolic condition documented in MDR before the spill (October 1, 2007—April 20, 2010) using the same case definition as a post-DWH incident case. We excluded prevalent cases from each analysis of the corresponding condition. To avoid data sparsity issues, we only retained outcomes for which there were at least 9 incident cases per exposure group during the overall study follow-up period. This resulted in evaluating a smaller number of outcomes in the within-responder comparisons than in the spill responder vs. non-responder comparisons.

### Calculation of Person-Time

The responder vs. non-responder person-time calculation including all active duty cohort members has been described previously [23]. Briefly, the follow-up time started on the latter of two dates, April 20, 2010 or the USCG entry date. Responders contributed events and person-time as non-responders until the first day of their DWH deployment. Because some responders may have sought care outside of the MHS during deployment (e.g., at BP mobile clinics) and such health encounters may not have been recorded in the MHS in a systematic way, we excluded responder events and person-time during deployment from the study observational period. Responders contributed events and person-time to the responder group starting on the day after their deployment ended. The end of follow-up time for all study comparisons was the earliest of: 1) the date of becoming an incident case of a particular endocrine/metabolic condition, 2) the end of the follow-up period (September 30, 2015), or 3) the USCG exit date.

### Statistical Analyses

The main analyses included ICD-9 codes in any diagnostic position. We used multivariable Cox proportional hazards regression to model associations between oil spill exposures and time to endocrine/metabolic incident events by calculating adjusted hazard ratios (aHRs) and 95% confidence intervals (95% CI). The within-responder associations (ever vs. never crude oil exposures and combined crude oil/dispersants exposure) were adjusted for age at baseline (years), sex (male, female), race (white, Black, other/unknown), and baseline cigarette smoking status (never, former, current, unknown), based on prior literature [16–19]. The responder vs. non-responder models were adjusted only for age, sex, and race because smoking information was not available for non-responders or for responders who did not complete an exit survey (*n* = 2,472, 41%). Among a subset of responders (98.7%) and non-responders (97.5%) with information on education, we additionally adjusted the main models for highest education attainment (< high school/high school, some college and above). Because there was virtually no change in the estimates after additional adjustment for education, to preserve model parsimony, we did not adjust the main analyses for education.

The assumption of proportionality of hazards was tested across the overall follow-up period (April 20, 2010/end of deployment through September 30, 2015) by calculating Pearson correlations between Schoenfeld residuals and follow-up time, applying methods described previously [24]. Briefly, when the proportionality assumption was violated (*p* < 0.05), we calculated aHRs and 95% CIs for two approximately equal-length sub-periods: 1) April 20, 2010/end of deployment through December 31, 2012 (hereafter referred to as “the earlier period”) and 2) January 1, 2013 through September 30, 2015 (hereafter referred to as “the later period”).

#### Sensitivity Analyses

To check the robustness of the main associations, we performed sensitivity analyses, which have been described previously [24]. Briefly, three sensitivity analyses were performed: 1) we restricted relevant ICD-9 codes to the first or second diagnostic position, rather than to any diagnostic position; 2) we excluded cohort members who were under more intensive periodic medical surveillance through enrollment in the USCG’s Occupational Medical Surveillance and Evaluation Program (OMSEP) at the time of the DWH oil spill or during the study follow-up period [25]; and 3) because tobacco smoke contains some of the same constituents as crude oil (i.e., benzene, PAHs, heavy metals), [26] we restricted the within-responder comparisons of ever vs. never crude oil inhalation exposure to responders who reported never smoking at baseline.

All analyses were performed in SAS Version 9.4 (SAS Institute, Cary, NC, USA).

## Results

### Baseline Population Characteristics

Baseline characteristics of the four groups of active duty DWH-CG Cohort members are presented in Table 1: 1) all non-responders (*n* = 39,230), 2) all responders (*n* = 5,964), 3) responders with any survey data (*n* = 3,492), and 4) responders without survey data (*n* = 2,472).

**Table 1:** Characteristics of active duty members of the Deepwater Horizon Oil Spill Coast Guard Cohort

Characteristic	**Non-responders (*N* = 39,260)**	**Responders (*N* = 5,964)**	**Responders with survey (*N* = 3,492)**	**Responders with no survey (*N* = 2,472)**
**Age (years)**				
Mean (SD)	30.3 (8.2)	30.7 (7.6)	30.9 (7.6)	30.5 (7.6)
**Sex, n (%)**				
Male	33,517 (85.4%)	5,238 (87.8%)	3,028 (86.7%)	2,210 (89.4%)
Female	5,743 (14.6%)	726 (12.2%)	464 (13.3%)	262 (10.6%)
**Race, n (%)**				
White	30,209 (76.9%)	4.630 (77.6%)	2,703 (77.4%)	1,927 (78.0%)
Black	2,183 (5.6%)	304 (5.1%)	167 (4.8%)	137 (5.5%)
Asian, AI/AN, NH/PI	1,541 (3.9%)	240 (4.0%)	153 (4.4%)	87 (3.5%)
Other	2,111 (5.4%)	300 (5.0%)	178 (5.1%)	122 (4.9%)
Unknown	3,216 (8.2%)	490 (8.3%)	291 (8.3%)	199 (8.1%)
**Military rank, n (%)**				
Junior enlisted (E1-E5)	22,191 (56.5%)	3,020 (50.6%)	1,696 (48.6%)	1,324 (53.6%)
Senior enlisted (E6-E10)	10,106 (25.7%)	1,454 (24.4%)	868 (24.9%)	586 (23.7%)
Officer (O1-O10, W2-W4)	6,963 (17.8%)	1,490 (25.0%)	928 (26.5%)	562 (22.7%)
**Highest education, n (%)**				
High school or less	27,401 (69.8%)	3,893 (65.3%)	2,242 (64.2%)	1,651 (66.8%)
Some college or higher*	10,862 (27.7%)	1,990 (33.4%)	1,203 (34.5%)	787 (31.8%)
Other or not indicated	997 (2.5%)	81 (1.3%)	47 (1.3%)	34 (1.4%)
**Smoking status, n (%)**				
Never	–	–	1,888 (54.1%)	–
Former	–	–	521 (14.9%)	–
Current	–	–	786 (22.5%)	–
Missing	–	–	297 (8.5%)	–
**Follow-up time (years)**				
Median	5.5	5.1	5.1	5.2

Abbreviations: *AI* American Indian, *AN* Alaska Native, *NH* Native Hawaiian, *PI* Pacific Islander

^*^Some college or higher includes technical school, bachelors, masters, and doctoral degree

The mean baseline age was approximately 30 years, irrespective of responder/non-responder status or survey completion status. Cohort members were largely male (> 85%) and white (~ 77%). The proportion of junior enlisted cohort members was the highest among non-responders (56.5%) and the lowest among responders with survey data (48.6%). A higher proportion of non-responders (69.8%) than responders (65.3%) had been educated at most through high school. Among responders who completed a post-deployment survey, the majority (54.1%) reported never smoking, 14.9% were former smokers, 22.5% were current smokers, while smoking status of the remaining 8.5% was unknown. The median follow-up time was 5.45 years for non-responders and 5.08 years for responders. By the end of the study follow-up, 36.8% of non-responders and 32.5% of responders had exited the USCG.

### Responder vs. Non-Responder Comparisons

The age-, sex-, and race-adjusted HRs for incident endocrine and metabolic conditions, comparing all active duty responders to non-responders, are presented in Table 2. The proportionality of hazards assumption over the study follow-up period (2010–2015) was violated for *dysmetabolic syndrome X* (currently referred to as metabolic syndrome) (*p* < 0.05), so the analyses for this outcome were conducted separately in the earlier (2010–2012) and in the later time period (2013–2015) (see Table 2 footnote). Compared to non-responders, responders had a significantly elevated risk for *dysmetabolic syndrome X* in the earlier time period (aHR = 2.07, 95% CI: 1.22–3.51), but not in the later time period (aHR = 0.93, 95% CI: 0.36–2.39), although the aHR during 2013–2015 was based on only 5 cases among responders (see Table 2 footnote). Where the proportionality of hazards assumption was not violated, responders had elevated risks for an enlarged thyroid gland condition *simple and unspecified goiter* (aHR = 2.09, 95% CI: 1.29–3.38) and *disorders of lipid metabolism* (aHR = 1.09, 95% CI: 1.00–1.18), including a high lipids condition *other and unspecified hyperlipidemia* (aHR = 1.10, 95% CI: 1.01–1.21). There was also an elevation in risk for the subcategory under *disorders of lipid metabolism* of *pure hypercholesterolemia* (aHR = 1.20, 95% CI: 0.98–1.48) and a reduction in risk for *diabetes mellitus* (aHR = 0.72, 95% CI: 0.50–1.03) among responders, although neither association was statistically significant. For the other conditions, associations were predominantly null. Additional adjustment for education did not meaningfully change any of the aHR estimates (Supplemental Table 2).

**Table 2:** Risk of endocrine/metabolic conditions comparing active duty Deepwater Horizon Oil Spill Coast Guard Cohort responders to non-responders, 2010–2015

	**Responder (*N* = 5,964)**		**Non-responder (*N* = 39,260)**		
**Condition (ICD-9 code)**	**N**	**Person Years**	**N**	**Person Years**	aHR* (95% CI)
Thyroid disorders combined (240–242,244–246)	99	26,049	688	174,061	1.02 (0.82–1.26)
Simple and unspecified goiter (240)	22	26,501	75	177,425	**2.09 (1.29–3.38)**
Non-toxic nodular goiter (241)	27	26,456	206	177,023	0.89 (0.60–1.33)
Acquired hypothyroidism (244)	58	26,246	419	175,311	1.00 (0.76–1.32)
Thyroiditis (245)	16	26,512	89	177,445	1.22 (0.72–2.08)
Other disorders of thyroid (246)	27	26,486	137	177,342	1.33 (0.88–2.01)
Diabetes mellitus (250)	32	26,437	299	176,557	0.72 (0.50–1.03)
Disorders of the pituitary gland and its hypothalamic control (253)	16	26,531	75	177,567	1.39 (0.81–2.39)
Disorders of lipid metabolism (272)	646	22,775	4,082	154,508	**1.09 (1.00–1.18)**
Pure hypercholesterolemia (272.0)	110	26,143	612	174,738	1.20 (0.98–1.48)
Pure hyperglyceridemia (272.1)	78	26,247	555	175,515	0.93 (0.73–1.18)
Mixed hyperlipidemia (272.2)	45	26,346	297	176,535	1.00 (0.73–1.37)
Other and unspecified hyperlipidemia (272.4)	565	23,376	3,532	158,316	**1.10 (1.01–1.21)**
Gout (274)	46	26,426	256	176,654	1.17 (0.85–1.60)
Dysmetabolic syndrome X (277.7)	23	26,514	107	177,506	1.64 (1.04–2.59)**
Overweight, obesity and other hyperalimentation (278)	448	24,316	3,390	162,998	0.95 (0.86–1.05)
Overweight and obesity (278.0)	447	24,319	3,376	163,088	0.95 (0.86–1.05)
Obesity, unspecified (278.00)	229	25,627	1,634	171,692	0.98 (0.85–1.13)
Overweight (278.02)	288	25,246	2,090	169,114	0.99 (0.87–1.12)
Abnormal weight gain (783.1)	29	26,470	233	176,969	0.88 (0.60–1.30)
Abnormal glucose tolerance test (790.21)	110	26,127	852	174,651	0.89 (0.73–1.08)
Impaired fasting glucose (790.21)	46	26,362	340	176,408	0.91 (0.67–1.24)

**Bold** indicative of statistical significance

^*^Models adjusted for age, sex, and race

^**^Because of the proportionality of hazards assumption violation for *dysmetabolic syndrome X* during 2010–2015 (Schoenfeld p < 0.05), results from sub-period analyses were: 2010–2012: N_responder_ = 18, N_non-responder_ = 75, aHR = 2.07, 95% CI: 1.22–3.51 and 2013–2015: N_responder_ = 5, N_non-responder_ = 32, aHR 0.93, 95% CI: 0.36–2.39

The sensitivity analysis where we refined endocrine and metabolic case definitions by restricting incident cases to those with ICD-9 codes in either the first or the second diagnostic position, instead of in *any* diagnostic position, is presented in Supplemental Table 3. The patterns and magnitudes of associations were generally similar to those in the main analysis presented in Table 2. The elevation in risk for *simple and unspecified goiter* among responders persisted (aHR = 1.93, 95% CI: 1.14–3.26). In this sensitivity analysis, the proportionality of hazards assumption was not violated for *dysmetabolic syndrome X* and the risk among responders was significantly elevated (aHR = 1.92, 95% CI: 1.10–3.36) in the overall time period. However, the proportionality of hazards assumption was violated for the subcategory under *disorders of lipid metabolism* of *other and unspecified hyperlipidemia*; the risk for this condition was elevated but not statistically significant in the earlier time period (aHR = 1.11, 95% CI: 0.99–1.25), but null in the later time period (aHR = 0.94, 95% CI: 0.77–1.14).

**Table 3:** Risk of endocrine/metabolic conditions among active duty Deepwater Horizon Oil Spill Coast Guard Cohort responders reporting ever vs. never exposure to crude oil inhalation, 2010–2015

	**Oil Inhalation Ever (*N* = 1,068)**		**Oil Inhalation Never (*N* = 2,424)**		
**Condition (ICD-9 code)**	**N**	**Person Years**	**N**	**Person Years**	aHR* (95% CI)
Thyroid disorders combined (240–242,244–246)	16	4,822	47	10,565	1.00 (0.56–1.79)
Acquired hypothyroidism (244)	10	4,848	31	10,639	0.90 (0.44–1.87)**
Disorders of lipid metabolism (272)	139	4,095	267	9,262	**1.24 (1.00–1.53)**
Pure hypercholesterolemia (272.0)	32	4,797	44	10,655	**1.71 (1.08–2.72)**
Pure hyperglyceridemia (272.1)	21	4,826	26	10,701	1.81 (1.01–3.25)**
Mixed hyperlipidemia (272.2)	12	4,834	22	10,751	1.30 (0.63–2.65)
Other and unspecified hyperlipidemia (272.4)	119	4,235	238	9,499	1.20 (0.96–1.51)
Gout (274)	12	4,873	21	10,778	1.37 (0.66–2.84)
Overweight, obesity and other hyperalimentation (278)	97	4,432	192	9,946	1.19 (0.93–1.53)
Overweight and obesity (278.0)	97	4,432	191	9,949	1.20 (0.94–1.54)
Obesity, unspecified (278.00)	59	4,674	95	10,464	**1.52 (1.09–2.13)**
Overweight (278.02)	56	4,638	118	10,334	1.13 (0.82–1.56)
Abnormal weight gain (783.1)	10	4,873	9	10,787	**2.60 (1.04–6.55)**
Abnormal glucose tolerance test (790.2)	19	4,845	59	10,588	0.81 (0.48–1.37)

**Bold** indicative of statistical significance

^*^Models adjusted for age, sex, race, and smoking

^**^ Because of the proportionality of hazards assumption violation for *acquired hypothyroidism* and for *pure hyperglyceridemia* during 2010–2015 (Schoenfeld *p* < 0.05), results from sub-period analyses were: 2010–2012: N_oil inhal ever_ = 4, N_oil inhal never_ = 18, aHR = 0.62, 95% CI: 0.20–1.86 and 2013–2015: N_oil inhal ever_ = 6, N_oil inhal never_ = 13, aHR 1.30, 95% CI: 0.48–3.51 and 2010–2012: N_oil inhal ever_ = 10, N_oil inhal never_ = 20, aHR = 1.09, 95% CI: 0.50–2.35 and 2013–2015: N_oil inhal ever_ = 11, N_oil inhal never_ = 6, aHR 4.31, 95% CI: 1.55–11.98, respectively

After exclusion of 1,114 (2.8%) non-responders and 236 (4.0%) responders who were enrolled in the USCG’s surveillance program, OMSEP, during the spill and the study follow-up period (Supplemental Table 4), the general magnitude and patterns of risk remained similar to the main analysis.

**Table 4:** Risk of endocrine/metabolic conditions among active duty DWH-CG Cohort responders reporting exposure to crude oil only (*N* = 1,351), both crude oil and dispersant (*N* = 448) vs. neither exposure (*N* = 1,283), 2010–2015

Condition (ICD-9 code)	N	Person Years	aHR* (95% CI)
**Thyroid disorders combined (240–242,244–246)**			
Neither	26	5,644	1.00
Oil only	19	5,985	0.90 (0.49–1.63)
Both	9	1,996	1.37 (0.63–2.97)
**Disorders of lipid metabolism (272)**			
Neither	148	4,938	1.00
Oil only	157	5,112	1.05 (0.84–1.32)
Both	68	1,691	**1.44 (1.08–1.93)**
**Pure hypercholesterolemia (272.0)**			
Neither	25	5,688	1.00
Oil only	36	6,001	1.46 (0.87–2.45)
Both	14	1,990	1.71 (0.88–3.31)
**Pure hyperglyceridemia (272.1)**			
Neither	12	5,722	1.00
Oil only	21	6,029	1.50 (0.74–3.07)
Both	9	2,008	1.91 (0.80–4.56)
**Other and unspecified hyperlipidemia (272.4)**			
Neither	129	5,072	1.00
Oil only	140	5,258	1.07 (0.84–1.37)
Both	59	1,750	**1.42 (1.04–1.93)**
**Overweight, obesity and other hyperalimentation (278)**			
Neither	98	5,288	1.00
Oil only	114	5,602	1.13 (0.86–1.48)
Both	46	1,849	1.39 (0.98–1.99)
**Overweight and obesity (278.0)**			
Neither	97	5,291	1.00
Oil only	114	5,602	1.14 (0.87–1.50)
Both	46	1,849	1.41 (0.99–2.01)
**Obesity, unspecified (278.00)**			
Neither	53	5,576	1.00
Oil only	51	5,900	0.94 (0.64–1.39)
Both	25	1,963	1.40 (0.87–2.27)
**Overweight (278.02)**			
Neither	61	5,506	1.00
Oil only	70	5,841	1.14 (0.80–1.61)
Both	29	1,910	1.46 (0.94–2.29)

**Bold** indicative of statistical significance

^*^Models adjusted for age, sex, race, and smoking

### Within-Responder Comparisons: Crude Oil Inhalation

Table 3 depicts age-, sex-, race-, and smoking-adjusted HRs and 95% CIs, comparing active duty responders who reported *ever* exposure to crude oil via inhalation to responders who reported *never* crude oil inhalation exposure. In the overall follow-up period, where the proportionality of hazards assumption was not violated, we observed elevated risks for *disorders of lipid metabolism* (aHR = 1.24, 95% CI: 1.00–1.53) and its subcategory of *pure hypercholesterolemia* (aHR = 1.71, 95% CI: 1.08–2.72), the subcategory under *overweight, obesity and other hyperalimentation* of *obesity, unspecified* (aHR = 1.52, 95% CI: 1.09–2.13), and *abnormal weight gain* (aHR = 2.60, 95% CI: 1.04–6.55). There were also elevated risks for the group of conditions *overweight, obesity and other hyperalimentation* (aHR = 1.19) and its subcategory of *overweight and obesity* (aHR = 1.20), and the subcategory, *under disorders of lipid metabolism* of *other and unspecified hyperlipidemia* (aHR = 1.20), although the CIs for those three conditions included unity. For two of the conditions, *acquired hypothyroidism* and the subcategory under *disorders of lipid metabolism* of *pure hyperglyceridemia*, the proportionality of hazards assumption was violated (*p* < 0.05), thus, we performed period-specific analyses. An insufficient number of incident cases of *acquired hypothyroidism* among the exposed precluded us from meaningfully evaluating the associations in the earlier and later time periods (Table 3 footnote). Oil inhalation exposure was not significantly associated with *pure hyperglyceridemia* in the earlier time period (aHR = 1.09, 95% CI: 0.50–2.35), while the *pure hyperglyceridemia* risk was significantly elevated in the later time period (aHR = 4.31, 95% CI: 1.55–11.98), although based on a small number of incident cases (11 among exposed and 6 among non-exposed). Additional adjustment for education did not appreciably change any of the estimates (Supplemental Table 5).

The sensitivity analysis excluding 55 (5.1%) responders reporting oil inhalation exposure and 95 (3.9%) oil inhalation non-exposed responders who were enrolled in OMSEP during the spill and the study follow-up period (Supplemental Table 6) yielded similar results to the main analysis. In the sensitivity analysis restricted to the 54.1% of responders who reported never smoking (Supplemental Table 7), patterns of risks were similar to the main analysis in Table 3, although we were unable to evaluate associations for *acquired hypothyroidism, pure hyperglyceridemia, mixed hyperlipidemia, gout, and abnormal weight gain* due to limited numbers of incident cases. Nevertheless, compared to the main analysis, the risks for *disorders of lipid metabolism* (aHR = 1.55, 95% CI: 1.17–2.06) and its subcategory *other and unspecified hyperlipidemia* (aHR = 1.47, 95% CI: 1.09–2.00) strengthened.

### Within-Responder Comparisons: Other Crude Oil Exposure Metrics

Results for the ever vs. never crude oil exposure via direct skin contact are presented in Supplemental Table 8. The associations between reporting the direct skin contact exposure and overweight and obesity outcomes were slightly stronger than for the crude oil inhalation metric presented in Table 3; the risks were significantly elevated for o*verweight, obesity and other hyperalimentation* (aHR = 1.37, 95% CI: 1.03–1.82) and its subcategories of *overweight and obesity* (aHR = 1.38, 95% CI: 1.04–1.83) and *obesity, unspecified* (aHR = 1.56, 95% CI: 1.07–2.27). Similarly, the associations between the direct skin contact exposure and *disorders of lipid metabolism* (aHR = 1.53, 95% CI: 1.21–1.95) and its subcategory of *other and unspecified hyperlipidemia* (aHR = 1.59, 95% CI: 1.23–2.04) were slightly stronger than for the crude oil inhalation metric. Due to the small number of incident cases (*n* < 9) for the outcomes of *acquired hypothyroidism*, the subcategories under *disorders of lipid metabolism* of *pure hyperglyceridemia* and *mixed hyperlipidemia, gout,* and *abnormal weight gain* among those reporting the direct skin contact exposure, we were unable to compare those associations to the main ones presented in Table 3.

### Within-Responder Comparisons: Combined Crude Oil and Dispersants Exposure

The adjusted associations of self-reported exposures to “oil only” and to both crude oil and dispersants (“both”) compared to neither exposure are presented in Table 4. The proportionality of hazards assumption was not violated for any of the outcomes in the overall follow-up period. The associations for all of the endocrine and metabolic conditions followed the same pattern of greater magnitude of risk among responders reporting exposure to both crude oil and dispersants (vs. neither) than among responders reporting “oil only” (vs. neither) exposure. The risk of *disorders of lipid metabolism* was significantly elevated for the exposure to both crude oil and dispersants (aHR = 1.44, 95% CI: 1.08–1.93), but null for the “oil only” vs. neither comparison (aHR = 1.05, 95% CI: 0.84–1.32). We observed similar associations for a subcategory of the *disorders of lipid metabolism*, the *other and unspecified hyperlipidemia* (i.e., aHR _both vs. neither_ = 1.42, 95% CI: 1.04–1.93, aHR _oil only vs. neither_ = 1.07, 95% CI: 0.84–1.37). Although not statistically significant, there was an increased risk of the subcategory of *overweight, obesity and other hyperalimentation* of *overweight and obesity* for the exposure to both crude oil and dispersants (aHR = 1.41, 95% CI: 0.99–2.01), and a smaller elevated risk for the “oil only” exposure (aHR = 1. 14, 95% CI: 0.87–1.50). The associations for the remaining endocrine and metabolic outcomes followed the same pattern.

## Discussion

In our cohort study of young and generally healthy active duty USCG service members with universal military healthcare coverage, we found that the oil spill response, as well as self-reported exposures to crude oil and to combined crude oil and dispersants during the spill cleanup, were associated with increased risk for diagnoses of several endocrine and metabolic conditions up to five and a half years following the *Deepwater Horizon* oil spill. Although a crude measure of exposure, responding vs. not responding to the spill was associated with up to two-fold increased risks of being diagnosed with an enlarged thyroid gland condition *simple and unspecified goiter*, *disorders of lipid metabolism* and its subcategory, a high cholesterol condition *other and unspecified hyperlipidemia*, as well as *dysmetabolic syndrome X*, also known as metabolic syndrome. Compared to non-exposed responders, those who reported crude oil exposure via different routes (e.g., inhalation, direct skin contact) were at increased risk for being diagnosed with a number of conditions related to elevated lipids and obesity/abnormal weight gain. Risk estimates for endocrine and metabolic conditions were generally higher in magnitude among responders reporting exposure to both crude oil and dispersants compared to those reporting neither exposure than among responders reporting exposure to crude oil only (vs. neither exposure). Patterns of risks remained similar across a range of sensitivity analyses.

To our knowledge, this is the only study that assessed multiple clinical endocrine and metabolic outcomes following an oil spill response. A recent GuLF Study investigation reported a modest association between increasing levels of estimated total hydrocarbon exposure and incident type 2 diabetes risk among a subsample of overweight participants [risk ratio (RR)_0.30–0.99 vs. <0.30 ppm_ = 0.99, 95% CI: 0.37–2.69, RR_1.00–2.99 vs. <0.30 ppm_ = 1.46, 95% CI: 0.54–3.92, RR_≥3.00 vs. <0.30 ppm_ = 2.11, 95% CI: 0.78–5.74, p-trend = 0.03] [19]. In our study, responders had a reduced but not statistically significant risk of diabetes compared to non-responders (aHR = 0.72, 95% CI: 0.50–1.03). The number of incident diabetes cases was too small among responders with survey data to investigate associations between crude oil exposures and diabetes risk. Due to the small number of incident diabetes cases in our population, restricting our sample to overweight individuals for comparison to the GuLF Study findings was not feasible. The demographic differences between the GuLF Study subsample and our study would also make the comparison of diabetes risk across the studies challenging, given that the majority of the GuLF Study participants (> 78%) were older than an average cohort member in our study (30 years) and 50% of the GuLF Study population was non-white. Our finding of reduced diabetes risk may be due to chance, especially because we observed elevated risks for conditions that are risk factors for developing type 2 diabetes (e.g., obesity).

Sub-clinical endocrine system-related outcomes have been previously studied following the 2002 *Prestige* oil spill in a form of plasma prolactin and cortisol levels as potential biomarkers of endocrine toxicity [15–18]. Those studies reported altered prolactin and cortisol levels among exposed cleanup participants acutely and up to seven years post-spill. While we did not have laboratory values for our cohort members, we evaluated ICD-9 diagnostic codes for endocrine conditions that are associated with alterations in prolactin and cortisol (i.e., hyperprolactinemia or galactorrhea not associated with childbirth); however, we did not include those conditions in the analysis due to low number of cases (i.e., < 5 among responders, Supplemental Table 1). Therefore, we were unable to directly compare our findings to the studies following the *Prestige* oil spill. Nevertheless, given that obesity is associated with elevated cortisol levels [27], our finding of elevated obesity risk among responders reporting crude oil exposures (via inhalation and direct skin contact) is in agreement with the prospective findings from the *Prestige* spill of mean cortisol levels being significantly higher among the exposed cleanup workers than among the non-exposed group seven years post-spill [18]. Additionally, total urinary PAH metabolites and naphthalene metabolites have been associated with higher body mass index (BMI), waist circumference, and obesity in children [28] and the highest quartiles of 2-OH Na and sum of PAH metabolites have been associated with obesity in a general adult population [29]. One meta-analysis showed significant positive associations between naphthalene, phenanthrene, and total OH-PAH metabolites and risk of obesity, with the pooled odds ratio (OR) and 95% CI estimates at 1.43 (1.07 – 1.90), 1.54 (1.18 – 2.02), and 2.29 (1.32 – 3.99), respectively [30].

Our findings of increased risk of *simple and unspecified goiter* associated with oil spill exposures are novel and have not been previously evaluated; however, there has been evidence of positive associations between urinary metabolites of PAHs, a constituent of crude oil, and nodular goiter [31]. In a case–control study in a non-occupationally exposed population, participants with urinary concentrations in the highest tertiles of two common PAHs, 2-hydroxyfluorene (2-OH FLU) and 1-hydroxyphenanthrene (1-OH PHE), had significantly higher risk of nodular goiter [31]. There are several mechanisms that may contribute to these findings. First, crude oil constituents, such as PAHs, have been shown to contribute to oxidative stress in vivo, [32, 33] and increased oxidative DNA damage has been observed in patients with goiter [34]. Additionally, thyroid hormones have been shown to be affected by exposure to crude oil [35] and thyroid hormone levels, particularly triiodothyronine (T3), have been associated with goiter [36]. Further study is needed to better understand this observed relationship.

Blood lipid profiles have been shown to be affected by PAH exposure, supporting our findings. One study found that high urinary total hydroxyphenanthrene (ΣOHPh) concentrations were associated with increased low-density lipoprotein cholesterol (LDL-C) in one non-occupationally exposed population [37]. Compared with participants who had low urinary ΣOHPh, those with high levels had an average increase of 0.137 mmol/l for total cholesterol and 0.129 mmol/l for LDL-C over 6 years of follow-up in that study. In other studies within the general population, compared to the lowest tertile of urinary PAH metabolites, increased risk of high total cholesterol was observed among those in the highest tertiles for 1-OH Na, 1-OH PHE, 9-hydroxyfluorene (9-OH FLU), and 4-hydroxyphenanthrene (4-OH PHE) and participants in the highest tertiles of 1-OH Na and 2-hydroxyfluorene (2-OH FLU) had higher risk for high LDL-C [38]. Additionally, an elevated ratio of triglycerides to high-density lipoprotein cholesterol (TG/HDL-C) was found among those in the highest quartiles of benzene exposure compared to those in the lowest quartile [39]. Similar findings have been found in occupationally-exposed populations, specifically chimney sweeps [40]. Elevated triglycerides [prevalence ratio (PR), 95% CI: 1.19, 1.09 – 1.30] and reduced high-density lipoprotein cholesterol (HDL-C) (PR, 95% CI: 1.11, 1.03–1.20) have been found with higher exposure to mixtures of PAHs in the National Health and Nutrition Examination Survey (NHANES) 2001–2012 population [41]. The mechanisms behind the relationships between crude oil and its constituents with dyslipidemia is unclear. It has been suggested that PAHs could activate the aryl hydrocarbon receptor (AhR) signaling pathway, which may lead to abnormal expression of cytochrome P450 and lead to the development of dyslipidemia [42, 43].

Metabolic syndrome, or dysmetabolic syndrome X, has been positively associated with urinary PAH concentrations in the NHANES 2003–2016 population and 2001–2012 population [41, 44]. It has been suggested that PAHs may interfere with the endocrine system through activation of peroxisome proliferator-activated receptors (PPARs) [45]. Murine studies have shown differences in lipid profiles following benzopyrene exposure, [46] and epidemiological studies have shown associations between PAH concentrations and increased HbA1c [47]. Given that metabolic syndrome is characterized by the coexistence of several factors that increase the risk of cardiovascular disease (e.g., high blood pressure, hyperglycemia, dyslipidemia) and that crude oil contains many constituents, many mechanisms may drive the observed associations. Additionally, a study among coke oven workers found smoking status to be an effect modifier in the relationship between urinary levels of the PAHs 1-OH Na and 2-OH FLU and metabolic syndrome [48]. Due to small counts, we could not evaluate metabolic syndrome risk among never-smoking active duty DWH responders, making it difficult to draw comparisons between studies.

Previous epidemiologic studies have reported that oil spill response exposures were associated with increased risk for longer-term cardiovascular outcomes including hypertension, palpitations, and self-reported myocardial infarction or fatal coronary heart disease [24, 49–51]. While those studies did not assess any endocrine or metabolic outcomes, it is possible that some of the conditions for which we observed elevated risks, such as dyslipidemia, overweight and obesity, metabolic syndrome, and abnormal weight gain, could eventually lead to the development of cardiovascular disease as those conditions are risk factors for heart disease. In fact, a recent study of incident diagnoses of cardiovascular risk factors among active duty U.S. military members (Army, Air Force, Navy, Marine Corps, and Air Force) reported that obesity, hyperlipidemia, essential hypertension, abnormal blood glucose level, and diabetes mellitus were the most frequently diagnosed cardiovascular risk factors between 2007 and 2016 [52]. During the time period that overlapped with our study follow-up (2010–2015), the unadjusted annual incidence rates of obesity among active duty service members doubled, while the annual incidence rates of hyperlipidemia decreased by about 30% [52]. Because that study did not include USCG personnel, it is challenging to compare the reported incidence rates between our studies, in particular since the reported incidence of cardiovascular risk factors differed widely by service branch [52]. The 2018 DoD Health Related Behaviors Survey showed that active duty USCG members were ahead of the Healthy People 2020 weight goals for obesity (i.e., 15% prevalence in the USCG compared to the target goal of < 30.5%), although the prevalence had increased since 2015 [53]. Nevertheless, given that obesity rates almost doubled between 2007 and 2016 in the U.S. military, [52] compared to a 17% increase in the U.S. civilian adult population during the same time period, [54] the results of our study further illuminate the importance of modifying risk factors associated with obesity in the military, both traditional (e.g., diet, exercise) and non-traditional (e.g., exposure to toxic exposures such as crude oil and dispersants in our study). Studies have classified the COREXIT dispersants constituent, DOSS, as a probable obesogen by PPARγ transactivation [13, 14]. Although we did not find statistically significant evidence to suggest exposure to both crude oil and dispersants increased risk of *overweight, obesity and other hyperalimentation* as a group, there was non-statistically significant evidence of higher risk for the subcategories of *overweight and obesity*; *obesity, unspecified*; and *overweight*, with non-significantly elevated aHRs among responders reporting exposure to both rather than oil only. We did find evidence of elevated risk of *disorders of lipid metabolism* and its subcategory *other and unspecified hyperlipidemia* with exposure to both dispersants and crude oil, versus oil only compared to neither, which is in line with findings in murine studies [55, 56].

The present study has several strengths, including the large sample size of our cohort which consisted of all active duty USCG members at the time of the DWH oil spill. This large sample allowed us to assess the robustness of our findings through multiple sensitivity analyses. Having information on the USCG’s occupational surveillance program (OMSEP) throughout the entire study period for both non-responders and responders allowed us to exclude cohort members who had occupational exposures (e.g., benzene) that could have already put them at a higher risk for developing endocrine or metabolic disease. Another strength of our study was examining several metrics of exposure, including crude oil via different routes (e.g., inhalation, direct skin contact) and a combined exposure to crude oil and dispersants, which put the response to the DWH oil spill into a more realistic context of different exposure scenarios. To our knowledge, our study was the first to ascertain longer-term incident endocrine and metabolic disease outcomes from an objective and comprehensive database of health encounters using strict diagnostic case definitions; this allowed us to eliminate the potential for recall errors in disease ascertainment. Because we had access to data on health encounters before the DWH oil spill, we were able to exclude USCG members with pre-existing conditions and to evaluate incident endocrine and metabolic diagnoses. However, since our cohort was comprised of young and relatively healthy active duty service members, the likelihood of existing co-morbidities was low. Having medical encounter information from a universal healthcare system designed for equal access and coverage likely also reduced the potential for differential loss to follow-up of our cohort members.

Our study findings should be interpreted in light of several limitations. First, we did not have any occupational monitoring data to capture individual-level exposures to the DWH spill chemicals. Our spill-response metrics were based on self-report from post-deployment surveys, which could have introduced recall errors. However, the USCG responders completed those surveys relatively shortly post-deployment (i.e., a median of 1 day for Survey 1 and 185 days for Survey 2) and any potential recall error would likely be non-differential. Our comparison of all DWH Coast Guard responders to non-responders, which included the entire active duty cohort, evaluated the possible impact of deployment itself, and not of specific spill-related chemical or other exposures. We were unable to adjust this responder vs. non-responder comparison for smoking because smoking information was available only for responders with survey data; this could have resulted in an overestimation of some of our estimates given that DWH deployment was associated with smoking initiation [57] and smoking was a risk factor for incident metabolic and endocrine conditions. The risk for endocrine and metabolic outcomes could have been underestimated in this comparison of responders to non-responders due to a healthy worker effect (i.e., a healthy deployer bias). At the time of the DWH oil spill, the USCG did not have a centralized database of personnel who were not fit for deployment (due to conditions such as pregnancy, injury, or an active infectious disease). Therefore, we were unable to exclude non-responders who may have been deemed medically unfit for deployment. This could have affected up to 10% of our non-responder population based on recent U.S. military estimates, [58] a proportion similar to the USCG estimates of personnel with a deployment-limiting condition (personal communication with RADM Dana L. Thomas on March 14, 2021). However, including only incident diagnoses in our analyses should have reduced the potential for this healthy deployer bias. The risk for endocrine and metabolic outcomes could have also been underestimated for responders because we excluded responder events and person-time during deployment from the study observational period since care sought outside of the MHS during deployment at places such as mobile clinics would have not been recorded in the military records.

A lack of information on potentially confounding factors that were not routinely recorded in the MDR at the time of our study, such as BMI, [59] cholesterol levels, [60] diet, [61–64] smoking, [65, 66] and physical activity, [67, 68] limited our ability to perform additional statistical adjustments. The results presented should be considered in light of these possible known lifestyle risk factors; however, we do not expect that these unmeasured factors would have differed by exposure status at baseline. However, performance of blood testing is required in the MHS to identify conditions that we evaluated, including hyperlipidemia, elevated blood glucose, and diabetes mellitus [52]. Because we performed multiple comparisons across a range of exposures and outcomes, some of our results may be statistically significant due to chance. However, most of our significant findings were robust across several sensitivity analyses. Moreover, given the lack of research on longer-term endocrine and metabolic health outcomes among oil spill responders, our primary goal was to evaluate different patterns of risk, rather than to test any specific hypothesis. While our outcomes were defined using objectively ascertained military health encounter records, ICD coding can be subject to classification inaccuracies such as coder errors [69]. Some of the other outcomes that we examined, such as obesity, are prone to high levels of underdiagnosis [70]. Nevertheless, ICD coding is a reliable indicator of health diagnoses when interpreted with caution, and has been widely used in epidemiological research [69] and for military surveillance efforts [52]. To increase the diagnostic accuracy of ICD coding classifications in our own study, we used an incident case definition of at least one inpatient or two outpatient visits. In a sensitivity analysis, we further refined our case definitions by limiting the ICD-9 codes to the first or the second diagnostic position and observed similar results. Additionally, we were limited to military health encounter records up to 5.5 years post-DWH, which may not reflect the latency period of some endocrine and metabolic conditions, which are largely unknown. Because some members of the CG-DWH cohort who completed a questionnaire do not have full coverage healthcare by the MHS (e.g., Selected Reserve members; 38%) we had to limit this analysis to active duty responders (68%) who completed a questionnaire and exclude Selected Reserve members (32%). Active duty members of the CG-DWH cohort who completed a questionnaire had a slightly higher proportion of younger, lower rank, male personnel compared to their Selected Reserve counterparts. Additionally, active duty had a slightly higher proportion reporting ever being exposed to crude oil (56%) than Selected Reserve personnel (51%), and there was a higher proportion of Selected Reserve personnel in administrative response jobs (53%) than active duty (35%). Therefore, the active duty study population studied here may represent a slightly higher oil spill response exposures than the Selected Reserve responders who were excluded from this analysis. Lastly, because our population consisted of young and generally healthy active duty military members who were predominantly white and male, these findings may not be generalizable to other responder populations.

## Conclusions

In this large cohort study of active duty USCG service members with universal military healthcare coverage, we observed that *Deepwater Horizon* cleanup exposures were moderately associated with increased risks for several longer-term clinical endocrine and metabolic conditions. This study provides further evidence of the endocrine-disrupting potential of certain chemicals in crude oil and dispersants. Oil spill disasters will continue to occur as deep water exploration and drilling becomes more aggressive [2, 71] and offshore drilling regulations become less strict [2]. Oil spills will continue to affect the ecosystem, wildlife, and human health, particularly spills that occur in environmentally sensitive areas that are already threatened by climate change. In order to inform disaster preparedness officials on preventative and mitigation measures needed to support responders to future oil spill disasters, it is of essential public health importance to continue studying acute and long-term adverse health outcomes of oil spill response workers and individuals residing in communities affected by oil spills.

## Supplementary Information

Supplementary Material 1.

## Data Availability

No datasets were generated or analysed during the current study.

## Abbreviations

aHR: Adjusted hazard ratio
AhR: Aryl hydrocarbon receptor
BMI: Body mass index
CI: Confidence interval
DoD: Department of Defense
DOSS: Dioctyl sodium sulfosuccinate
DWH: Deepwater Horizon
DWH-CG Cohort Study: The Deepwater Horizon Coast Guard Cohort Study
EPA: Environmental Protection Agency
GuLF Study: The Gulf Long Term Follow-up Study
HDL-C: High-density lipoprotein cholesterol
HR: Hazard ratio
ICD: The International Classification of Diseases
IRB: The Institutional Review Board
LDL-C: Low-density lipoprotein cholesterol
MDR: The Military Health System Data Repository
MHS: The Military Health System
NHANES: National Health and Nutrition Examination Survey
OMSEP: Occupational Medical Surveillance and Evaluation Program
OR: Odds ratio
OSHA: The Occupational Safety and Health Administration
PAH: Polycyclic aromatic hydrocarbon
PPAR: Peroxisome proliferator activated receptor
PR: Prevalence ratio
RR: Risk ratio
TG/HDL-C: Ratio of triglycerides to high-density lipoprotein cholesterol
T3: Triiodothyronine
USCG: U.S. Coast Guard
USU: The Uniformed Services University
VOC: Volatile organic compound
1-OH PHE: 1-Hydroxyphenanthrene
1-OH Na: 1-Hydroxynaphthalene
2-OH FLU: 2-Hydroxyfluorene
4-OH PHE: 4-Hydroxyphenanthrene
9-OH FLU: 9-Hydroxyfluorene
ΣOHPh: Total hydroxyphenanthrene
