# Supplementary material for: Risk of longer-term endocrine and metabolic conditions in the Deepwater Horizon Oil Spill Coast Guard cohort study – five years of follow-up
Source: Environ Health. 2025 Mar 22;24:12. doi: 10.1186/s12940-025-01164-9 (PMC11929317; doi:10.1186/s12940-025-01164-9)
Supplement: Supplementary file 1 — Supplementary Material 1. [file 12940_2025_1164_MOESM1_ESM.docx]

| Supplemental Table 1. A full listing of ICD-9 diagnostic codes used to define endocrine and metabolic conditions in the Deepwater Horizon Oil Spill Coast Guard Cohort | | | |
| --- | --- | --- | --- |
| 3-digit  ICD-9 code(s) | 4-digit  ICD-9 code(s) | 5-digit  ICD-9 code | Condition Description |
| 240-242,244-246 |  |  | Thyroid disorders combined |
| 240 |  |  | Simple and unspecified goiter |
| 241 |  |  | Non-toxic nodular goiter |
| 242 |  |  | Thyrotoxicosis with or without goiter* |
| 244 |  |  | Acquired hypothyroidism |
| 245 |  |  | Thyroiditis |
| 246 |  |  | Other disorders of thyroid |
| 250 |  |  | Diabetes mellitus |
| 252 |  |  | Disorders of parathyroid gland* |
| 253 |  |  | Disorders of the pituitary gland and its hypothalamic control |
| 255 |  |  | Disorders of adrenal glands* |
| 272 |  |  | Disorders of lipid metabolism |
|  | 272.0 |  | Pure hypercholesterolemia |
|  | 272.1 |  | Pure hyperglyceridemia |
|  | 272.2 |  | Mixed hyperlipidemia |
|  | 272.4 |  | Other and unspecified hyperlipidemia |
|  | 272.8 |  | Other disorders of lipid metabolism |
|  | 272.9 |  | Unspecified disorder of lipid metabolism |
| 274 |  |  | Gout |
|  | 277.7 |  | Dysmetabolic syndrome X |
| 278 |  |  | Overweight, obesity and other hyperalimentation |
|  | 278.0 |  | Overweight and obesity |
|  |  | 278.00 | Obesity, unspecified |
|  |  | 278.01 | Morbid obesity* |
|  |  | 278.02 | Overweight |
|  |  | 278.03 | Obesity hypoventilation syndrome* |
|  | 253.1, 611.6 |  | Hyperprolactinemia or galactorrhea not associated with childbirth* |
|  | 783.1 |  | Abnormal weight gain |
|  | 790.2 |  | Abnormal glucose tolerance test |
|  |  | 790.21 | Impaired fasting glucose |
|  |  | 790.22 | Impaired glucose tolerance test (oral)* |

*Excluded from analyses because <10 cases per exposure group (responder or non-responder)

| Supplemental Table 2. Risk of endocrine/metabolic conditions comparing active duty DWH-CG Cohort responders to non-responders among 44,146 cohort members without missing education data (2010-2015) | | | | | | |
| --- | --- | --- | --- | --- | --- | --- |
|  | **Responder (N=5,883)** | | **Non-responder (N=38,263)** | |  |  |
| **Condition (ICD-9 code)** | **N** | **Person Years** | **N** | **Person Years** | **HR^1^ (95% CI)** | **HR^2^ (95% CI)** |
| Thyroid disorders combined (240-242,244-246) | 96 | 25,674 | 677 | 169,476 | 0.99 (0.80-1.23) | 1.00 (0.81-1.24) |
| Simple and unspecified goiter (240) | 22 | 26,124 | 74 | 172,808 | **2.10 (1.30-3.39)** | **2.17 (1.34-3.50)** |
| Non-toxic nodular goiter (241) | 27 | 26,079 | 203 | 172,413 | 0.90 (0.60-1.34) | 0.91 (0.61-1.36) |
| Acquired hypothyroidism (244) | 57 | 25,869 | 413 | 170,712 | 0.99 (0.75-1.30) | 0.99 (0.75-1.31) |
| Thyroiditis (245) | 16 | 26,134 | 88 | 172,828 | 1.22 (0.72-2.09) | 1.23 (0.72-2.10) |
| Other disorders of thyroid (246) | 26 | 26,109 | 134 | 172,724 | 1.30 (0.85-1.97) | 1.31 (0.86-2.01) |
| Diabetes mellitus (250) | 32 | 26,059 | 299 | 171,936 | 0.72 (0.50-1.03) | 0.74 (0.51-1.07) |
| Disorders of the pituitary gland and its hypothalamic control (253) | 15 | 26,154 | 74 | 172,948 | 1.31 (0.75-2.29) | 1.34 (0.76-2.33) |
| Disorders of lipid metabolism (272) | 641 | 22,426 | 4,045 | 150,032 | **1.08 (1.00-1.18)** | **1.10 (1.01-1.19)** |
| Pure hypercholesterolemia (272.0) | 109 | 25,772 | 611 | 170,118 | 1.19 (0.97-1.46) | 1.20 (0.97-1.47) |
| Pure hyperglyceridemia (272.1) | 77 | 25,875 | 548 | 170,915 | 0.92 (0.73-1.17) | 0.94 (0.74-1.19) |
| Mixed hyperlipidemia (272.2) | 45 | 26,968 | 296 | 171,919 | 1.00 (0.73-1.37) | 1.02 (0.74-1.40) |
| Other and unspecified hyperlipidemia (272.4) | 560 | 23,021 | 3,504 | 153,805 | **1.10 (1.00-1.20)** | **1.11 (1.01-1.21)** |
| Gout (274) | 45 | 26,050 | 253 | 172,040 | 1.15 (0.84-1.59) | 1.19 (0.86-1.63) |
| Dysmetabolic syndrome X (277.7) | 23 | 26,137 | 107 | 172,886 | **1.64 (1.04-2.59)^3^** | **1.70 (1.07-2.69)^4^** |
| Overweight, obesity and other hyperalimentation (278) | 443 | 23,962 | 3,334 | 158,552 | 0.95 (0.86-1.05) | 0.97 (0.88-1.07) |
| Overweight and obesity (278.0) | 442 | 23,965 | 3,320 | 158,642 | 0.95 (0.86-1.05) | 0.97 (0.88-1.07) |
| Obesity, unspecified (278.00) | 227 | 25,524 | 1,620 | 167,116 | 0.97 (0.85-1.12) | 1.01 (0.87-1.16) |
| Overweight (278.02) | 286 | 24,877 | 2,048 | 164,630 | 0.99 (0.87-1.12) | 1.01 (0.89-1.14) |
| Abnormal weight gain (783.1) | 28 | 26,094 | 230 | 172,354 | 0.85 (0.58-1.27) | 0.88 (0.59-1.31) |
| Abnormal glucose tolerance test (790.2) | 110 | 25,749 | 845 | 170,049 | 0.90 (0.73-1.09) | 0.91 (0.74-1.11) |
| Impaired fasting glucose (790.21) | 46 | 25,984 | 339 | 171,789 | 0.91 (0.67-1.24) | 0.94 (0.69-1.28) |

^1^Models adjusted for age, sex, and race; ^2^ additionally adjusted for education (<high school/high school; some college and above)

^3^Because of the proportionality of hazards assumption violation, results from sub-period analyses were: 2010-2012: N_responder_=18, N_non-responder_=75, HR=2.07, 95% CI: 1.22-3.51 and 2013-2015: N_responder_ =5, N_non-responder_ =32, HR 0.93, 95% CI: 0.36-2.38.

^4^Because of the proportionality of hazards assumption violation, results from sub-period analyses were: 2010-2012: HR=2.12, 95% CI: 1.25-3.60 and 2013-2015: HR 0.99, 95% CI: 0.38-2.54.

Bold indicative of statistical significance.

| **Supplemental Table 3. Risk of endocrine/metabolic conditions comparing active duty DWH-CG Cohort responders to non-responders, 2010-2015; ICD-9 diagnostic codes in first or second diagnostic position** | | | | | |
| --- | --- | --- | --- | --- | --- |
|  | **Responder (N=5,964)** | | **Non-responder (N=39,260)** | |  |
| **Condition (ICD-9 code)** | **N** | **Person Years** | **N** | **Person Years** | **HR* (95% CI)** |
| Thyroid disorders combined (240-242,244-246) | 87 | 26,080 | 623 | 174,409 | 1.00 (0.80-1.25) |
| Simple and unspecified goiter (240) | 18 | 26,520 | 66 | 177,488 | **1.93 (1.14-3.26)** |
| Non-toxic nodular goiter (241) | 27 | 26,456 | 191 | 177,075 | 0.96 (0.64-1.44) |
| Acquired hypothyroidism (244) | 52 | 26,259 | 382 | 175,501 | 1.00 (0.74-1.33) |
| Thyroiditis (245) | 12 | 26,529 | 81 | 177,475 | 1.01 (0.55-1.86) |
| Other disorders of thyroid (246) | 21 | 26,506 | 103 | 177,472 | 1.41 (0.88-2.26) |
| Diabetes mellitus (250) | 30 | 26,447 | 263 | 176,704 | 0.76 (0.52-1.11) |
| Disorders of the pituitary gland and its hypothalamic control (253) | 13 | 26,536 | 65 | 177,612 | 1.29 (0.71-2.34) |
| Disorders of lipid metabolism (272) | 511 | 23,346 | 3,393 | 157,814 | 1.05 (0.95-1.15) |
| Pure hypercholesterolemia (272.0) | 69 | 26,275 | 371 | 175,744 | 1.28 (0.99-1.66) |
| Pure hyperglyceridemia (272.1) | 54 | 26,325 | 394 | 176,056 | 0.92 (0.69-1.23) |
| Mixed hyperlipidemia (272.2) | 35 | 26,361 | 219 | 176,868 | 1.05 (0.73-1.50) |
| Other and unspecified hyperlipidemia (272.4) | 450 | 23,841 | 2,946 | 161,075 | 1.06 (0.96-1.17)** |
| Gout (274) | 36 | 26,436 | 232 | 176,766 | 1.01 (0.71-1.44) |
| Dysmetabolic syndrome X (277.7) | 16 | 26,531 | 68 | 177,617 | **1.92 (1.10-3.36)** |
| Overweight, obesity and other hyperalimentation (278) | 293 | 24,974 | 2,235 | 167,019 | 0.98 (0.87-1.11) |
| Overweight and obesity (278.0) | 293 | 24,978 | 2,223 | 167,097 | 0.99 (0.88-1.12) |
| Obesity, unspecified (278.00) | 149 | 25,926 | 1,045 | 173,599 | 1.01 (0.85-1.20) |
| Overweight (278.02) | 183 | 25,624 | 1,297 | 171,698 | 1.07 (0.92-1.26) |
| Abnormal weight gain (783.1) | 24 | 26,486 | 179 | 177,155 | 0.95 (0.62-1.46) |
| Abnormal glucose tolerance test (790.2) | 72 | 26,278 | 508 | 175,920 | 0.98 (0.77-1.26) |
| Impaired fasting glucose (790.21) | 34 | 26,423 | 204 | 176,973 | 1.13 (0.78-1.62) |

*Models adjusted for age, sex, and race;

** Because of the proportionality of hazards assumption violation for *other and unspecified hyperlipidemia* during 2010-2015 (Schoenfeld p<0.05), results from sub-period analyses were: 2010-2012: N_responder_=338, N_non-responder_=2,244, HR=1.11, 95% CI: 0.99-1.25 and 2013-2015: N_responder_ =112, N_non-responder_ =702, HR 0.94, 95% CI: 0.77-1.14.

Bold indicative of statistical significance.

| **Supplemental Table 4. Risk of endocrine/metabolic conditions comparing active duty DWH-CG Cohort responders to non-responders after exclusion of 1,350 Occupational Medical Surveillance and Evaluation Program (OMSEP) enrollees; 2010-2015** | | | | | |
| --- | --- | --- | --- | --- | --- |
|  | **Responder (N=5,728)** | | **Non-responder (N=38,146)** | |  |
| **Condition (ICD-9 code)** | **N** | **Person Years** | **N** | **Person Years** | **HR* (95% CI)** |
| Thyroid disorders combined (240-242,244-246) | 96 | 24,875 | 674 | 168,122 | 1.02 (0.82-1.26) |
| Simple and unspecified goiter (240) | 22 | 25,316 | 73 | 171,433 | **2.16 (1.34-3.50)** |
| Non-toxic nodular goiter (241) | 26 | 25,273 | 204 | 171,032 | 0.87 (0.58-1.32) |
| Acquired hypothyroidism (244) | 57 | 25,064 | 412 | 169,341 | 1.01 (0.76-1.33) |
| Thyroiditis (245) | 16 | 25,326 | 89 | 171,441 | 1.23 (0.72-2.10) |
| Other disorders of thyroid (246) | 25 | 25,308 | 133 | 171,350 | 1.28 (0.83-1.96) |
| Diabetes mellitus (250) | 32 | 25,251 | 297 | 170,566 | 0.73 (0.50-1.05) |
| Disorders of the pituitary gland and its hypothalamic control (253) | 14 | 25,355 | 73 | 171,573 | 1.26 (0.71-2.24) |
| Disorders of lipid metabolism (272) | 622 | 21,757 | 3,963 | 149,148 | **1.09 (1.00-1.19)** |
| Pure hypercholesterolemia (272.0) | 108 | 24,970 | 601 | 168,781 | 1.21 (0.99-1.49) |
| Pure hyperglyceridemia (272.1) | 75 | 25,076 | 543 | 169,575 | 0.92 (0.72-1.17) |
| Mixed hyperlipidemia (272.2) | 43 | 25,180 | 294 | 170,554 | 0.98 (0.71-1.35) |
| Other and unspecified hyperlipidemia (272.4) | 546 | 22,316 | 3,424 | 152,867 | **1.11 (1.01-1.22)** |
| Gout (274) | 45 | 25,246 | 250 | 170,683 | 1.18 (0.86-1.63) |
| Dysmetabolic syndrome X (277.7) | 23 | 25,334 | 105 | 171,510 | **1.69 (1.07-2.68)**** |
| Overweight, obesity and other hyperalimentation (278) | 432 | 23,211 | 3,278 | 157,506 | 0.96 (0.87-1.06) |
| Overweight and obesity (278.0) | 431 | 23,214 | 3,264 | 157,596 | 0.96 (0.87-1.06) |
| Obesity, unspecified (278.00) | 221 | 24,475 | 1,590 | 165,865 | 0.98 (0.85-1.13) |
| Overweight (278.02) | 280 | 24,110 | 2,011 | 163,438 | 1.01 (0.89-1.14) |
| Abnormal weight gain (783.1) | 29 | 25,284 | 229 | 170,990 | 0.90 (0.61-1.33) |
| Abnormal glucose tolerance test (790.2) | 105 | 24,960 | 821 | 168,743 | 0.89 (0.73-1.09) |
| Impaired fasting glucose (790.21) | 44 | 25,187 | 325 | 170,456 | 0.92 (0.67-1.27) |

*Models adjusted for age, sex, and race;

** Because of the proportionality of hazards assumption violation for *dysmetabolic syndrome X* during 2010-2015 (Schoenfeld p<0.05), results from sub-period analyses were: 2010-2012: N_responder_=18, N_non-responder_=74, HR=2.12, 95% CI: 1.25-3.61 and 2013-2015: N_responder_ =5, N_non-responder_ =31, HR 0.97, 95% CI: 0.38-2.49.

Bold indicative of statistical significance.

| **Supplemental Table 5. Risk of endocrine/metabolic conditions among active duty DWH-CG Cohort responders reporting ever vs. never exposure to crude oil inhalation among 3,445 responders without missing education data (2010-2015)** | | | | | | |
| --- | --- | --- | --- | --- | --- | --- |
|  | **Oil inhalation ever (N=1,054)** | | **Oil inhalation never (N=2,391)** | |  |  |
| **Condition (ICD-9 code)** | **N** | **Person Years** | **N** | **Person Years** | **HR^1^ (95% CI)** | **HR^2^ (95% CI)** |
| Thyroid disorders combined (240-242,244-246) | 15 | 4,755 | 45 | 10,415 | 0.98 (0.54-1.78) | 0.95 (0.52-1.73) |
| Acquired hypothyroidism (244) | 10 | 4,780 | 30 | 10,488 | 0.94 (0.45-1.96)^3^ | 0.91 (0.44-1.90)^4^ |
| Disorders of lipid metabolism (272) | 138 | 4,032 | 264 | 9,122 | **1.24 (1.01-1.53)** | 1.22 (0.99-1.50) |
| Pure hypercholesterolemia (272.0) | 31 | 4,731 | 44 | 10,503 | **1.66 (1.04-2.66)** | **1.66 (1.04-2.66)** |
| Pure hyperglyceridemia (272.1) | 21 | 4,758 | 25 | 10,553 | **1.83 (1.02-3.31)^3^** | 1.73 (0.96-3.12)^4^ |
| Mixed hyperlipidemia (272.2) | 12 | 4,766 | 22 | 10,599 | 1.29 (0.63-2.64) | 1.34 (0.65-2.75) |
| Other and unspecified hyperlipidemia (272.4) | 118 | 4,172 | 236 | 9,353 | 1.21 (0.96-1.51) | 1.19 (0.95-1.49) |
| Gout (274) | 12 | 4,805 | 21 | 10,626 | 1.37 (0.66-2.83) | 1.28 (0.62-2.66) |
| Overweight, obesity and other hyperalimentation (278) | 96 | 4,366 | 190 | 9,802 | 1.19 (0.93-1.53) | 1.15 (0.90-1.48) |
| Overweight and obesity (278.0) | 96 | 4,366 | 189 | 9,805 | 1.20 (0.94-1.54) | 1.16 (0.90-1.49) |
| Obesity, unspecified (278.00) | 58 | 4,608 | 95 | 10,311 | **1.50 (1.07-2.10)** | **1.42 (1.01-1.98)** |
| Overweight (278.02) | 56 | 4,570 | 117 | 10,185 | 1.14 (0.82-1.58) | 1.12 (0.81-1.54) |
| Abnormal weight gain (783.1) | 9 | 4,807 | 9 | 10,635 | 2.33 (0.91-6.00) | 2.25 (0.87-5.81) |
| Abnormal glucose tolerance test (790.2) | 19 | 4,777 | 59 | 10,436 | 0.81 (0.48-1.36) | 0.81 (0.48-1.38) |

^1^Models adjusted for age, sex, race, and smoking; ^2^ additionally adjusted for education (<high school/high school; some college and above)

^3^Because of the proportionality of hazards assumption violation for *acquired hypothyroidism* and for *pure hyperglyceridemia* during 2010- 2015 (Schoenfeld p<0.05), results from sub-period analyses were: 2010-2012: N_oil inhal ever_=4, N_oil inhal never_=18, HR=0.62, 95% CI: 0.21-1.87 and 2013-2015: N_oil inhal ever_ =6, N_oil inhal never_ =12, HR 1.43, 95% CI: 0.52-3.93 and 2010-2012: N_oil inhal ever_=10, N_oil inhal never_=19, HR=1.10, 95% CI: 0.51-2.39 and 2013-2015: N_oil inhal ever_ =11, N_oil inhal never_ =6, HR 4.33, 95% CI: 1.55-12.04, respectively.

^4^Because of the proportionality of hazards assumption violation for *acquired hypothyroidism* and for *pure hyperglyceridemia* during 2010- 2015 (Schoenfeld p<0.05), results from sub-period analyses were: 2010-2012: HR=0.59, 95% CI: 0.20-1.80 and 2013-2015: HR 1.39, 95% CI: 0.51-3.84 and 2010-2012: HR=1.04, 95% CI: 0.48-2.25 and 2013-2015: HR 4.10, 95% CI: 1.47-11.43, respectively.

Bold indicative of statistical significance.

| **Supplemental Table 6. Risk of endocrine/metabolic conditions among active duty DWH-CG Cohort responders reporting ever vs. never exposure to crude oil inhalation after exclusion of 150 Occupational Medical Surveillance and Evaluation Program (OMSEP) enrollees; 2010-2015** | | | | | |
| --- | --- | --- | --- | --- | --- |
|  | **Oil inhalation ever (N=1,013)** | | **Oil inhalation never (N=2,329)** | |  |
| **Condition (ICD-9 code)** | **N** | **Person Years** | **N** | **Person Years** | **HR* (95% CI)** |
| Thyroid disorders combined (240-242,244-246) | 15 | 4,545 | 45 | 10,094 | 0.99 (0.54-1.80) |
| Acquired hypothyroidism (244) | 10 | 4,567 | 30 | 10,164 | 0.95 (0.46-1.98)** |
| Disorders of lipid metabolism (272) | 133 | 3,857 | 255 | 8,865 | **1.25 (1.01-1.54)** |
| Pure hypercholesterolemia (272.0) | 30 | 4,523 | 44 | 10,177 | 1.59 (0.99-2.56) |
| Pure hyperglyceridemia (272.1) | 19 | 4,551 | 26 | 10,227 | 1.67 (0.91-3.06)** |
| Mixed hyperlipidemia (272.2) | 12 | 4,563 | 20 | 10,283 | 1.44 (0.69-2.97) |
| Other and unspecified hyperlipidemia (272.4) | 116 | 3,981 | 227 | 9,085 | 1.24 (0.99-1.56) |
| Gout (274) | 12 | 4,597 | 21 | 10,300 | 1.38 (0.67-2.86) |
| Overweight, obesity and other hyperalimentation (278) | 96 | 4,170 | 182 | 9,509 | 1.26 (0.98-1.62) |
| Overweight and obesity (278.0) | 96 | 4,170 | 181 | 9,513 | 1.27 (0.99-1.63) |
| Obesity, unspecified (278.00) | 59 | 4,398 | 88 | 10,008 | **1.67 (1.19-2.35)** |
| Overweight (278.02) | 55 | 4,366 | 114 | 9,876 | 1.16 (0.84-1.61) |
| Abnormal weight gain (783.1) | 10 | 4,592 | 9 | 10,309 | **2.62 (1.04-6.58)** |
| Abnormal glucose tolerance test (790.2) | 17 | 4,568 | 56 | 10,125 | 0.76 (0.44-1.32) |

*Models adjusted for age, sex, race, and smoking;

** Because of the proportionality of hazards assumption violation for *acquired hypothyroidism* and for *pure hyperglyceridemia* during 2010-2015 (Schoenfeld p<0.05), results from sub-period analyses were: 2010-2012: N_oil inhal ever_=4, N_oil inhal never_=17, HR=0.68, 95% CI: 0.22-2.06 and 2013-2015: N_oil inhal ever_ =6, N_oil inhal never_ =13, HR 1.31, 95% CI: 0.48-3.53 and 2010-2012: N_oil inhal ever_=9, N_oil inhal never_=20, HR=1.00, 95% CI: 0.45-2.22 and 2013-2015: N_oil inhal ever_ =10, N_oil inhal never_ =6, HR 4.03, 95% CI: 1.42-11.47, respectively.

Bold indicative of statistical significance.

| **Supplemental Table 7. Risk of endocrine/metabolic conditions among never-smoking active duty DWH-CG Cohort responders reporting ever vs. never exposure to crude oil inhalation; 2010-2015** | | | | | |
| --- | --- | --- | --- | --- | --- |
|  | **Oil inhalation ever (N=555)** | | **Oil inhalation never (N=1,333)** | |  |
| **Condition (ICD-9 code)** | **N** | **Person Years** | **N** | **Person Years** | **HR* (95% CI)** |
| Thyroid disorders combined (240-242,244-246) | 11 | 2,547 | 24 | 5,858 | 1.53 (0.74-3.17) |
| Acquired hypothyroidism (244) | <9 | 2,563 | 14 | 5,902 | **--** |
| Disorders of lipid metabolism (272) | 77 | 2,182 | 136 | 5,156 | **1.55 (1.17-2.06)** |
| Pure hypercholesterolemia (272.0) | 17 | 2,539 | 27 | 5,924 | 1.84 (0.99-3.40) |
| Pure hyperglyceridemia (272.1) | <9 | 2,578 | 13 | 5,950 | **--** |
| Mixed hyperlipidemia (272.2) | <9 | 2,566 | 13 | 5,956 | **--** |
| Other and unspecified hyperlipidemia (272.4) | 65 | 2,243 | 123 | 5,288 | **1.47 (1.09-2.00)** |
| Gout (274) | <9 | 2,592 | 9 | 5,992 | **--** |
| Overweight, obesity and other hyperalimentation (278) | 49 | 2,348 | 99 | 5,575 | 1.25 (0.88-1.76) |
| Overweight and obesity (278.0) | 49 | 2,348 | 98 | 5,578 | 1.26 (0.89-1.79) |
| Obesity, unspecified (278.00) | 27 | 2,476 | 44 | 5,849 | 1.58 (0.97-2.56) |
| Overweight (278.02) | 30 | 2,465 | 63 | 5,751 | 1.20 (0.77-1.86) |
| Abnormal weight gain (783.1) | <9 | 2,585 | <9 | 5,999 | **--** |
| Abnormal glucose tolerance test (790.2) | 9 | 2,566 | 31 | 5,870 | 0.75 (0.36-1.59) |

*Models adjusted for age, sex, and race

Bold indicative of statistical significance.

| **Supplemental Table 8. Risk of endocrine/metabolic conditions among active duty DWH-CG Cohort responders reporting ever vs. never exposure to crude oil via direct skin contact, 2010-2015** | | | | | |
| --- | --- | --- | --- | --- | --- |
|  | **Direct skin contact ever (n=628)** | | **Direct skin contact never (n=2864)** | |  |
| **Condition (ICD-9 code)** | **N** | **Person Years** | **N** | **Person Years** | **HR* (95% CI)** |
| Thyroid disorders combined (240-242,244-246) | 12 | 2,764 | 51 | 12,623 | 1.35 (0.71-2.55) |
| Acquired hypothyroidism (244) | <9 | 2,779 | 33 | 12,708 | **-** |
| Disorders of lipid metabolism (272) | 90 | 2,325 | 316 | 11,032 | **1.53 (1.21-1.95)** |
| Pure hypercholesterolemia (272.0) | 18 | 2,746 | 58 | 12,707 | 1.60 (0.94-2.73) |
| Pure hyperglyceridemia (272.1) | <9 | 2,754 | 39 | 12,773 | **-** |
| Mixed hyperlipidemia (272.2) | <9 | 2,761 | 26 | 12,824 | **-** |
| Other and unspecified hyperlipidemia (272.4) | 80 | 2,387 | 277 | 11,347 | **1.59 (1.23-2.04)** |
| Gout (274) | <9 | 2,789 | 26 | 12,862 | **-** |
| Overweight, obesity and other hyperalimentation (278) | 63 | 2,534 | 226 | 11,844 | **1.37 (1.03-1.82)** |
| Overweight and obesity (278.0) | 63 | 2,534 | 225 | 11,847 | **1.38 (1.04-1.83)** |
| Obesity, unspecified (278.00) | 36 | 2,677 | 118 | 12,460 | **1.56 (1.07-2.27)** |
| Overweight (278.02) | 34 | 2,659 | 140 | 12,312 | 1.17 (0.80-1.71) |
| Abnormal weight gain (783.1) | <9 | 2,793 | 15 | 12,867 | **-** |
| Abnormal glucose tolerance test (790.2) | 11 | 2,754 | 67 | 12,679 | 0.95 (0.50-1.81) |

*Models adjusted for age, sex, race, and smoking

Bold indicative of statistical significance.

**Supplemental Figure 1**





Study population. Flow chart showing definition of the study population and reasons for exclusion.
